# Maria Adelaide brace in the management of Scheuermann’s Kyphosis

**DOI:** 10.1007/s43390-020-00225-y

**Published:** 2020-11-18

**Authors:** Andrea Piazzolla, Davide Bizzoca, Giuseppe Solarino, Marco Brayda-Bruno, Giuseppe Tombolini, Alessio Ariagno, Biagio Moretti

**Affiliations:** 1 Department of Basic Medical Sciences, Neuroscience and Sense Organs, Orthopaedic, Trauma and Spine Unit, UOSD Spinal Deformity Center, School of Medicine, University of Bari Aldo Moro, AOU Consorziale “Policlinico”, Piazza Giulio Cesare 11, 70100 Bari, Italy; 2 Orthopaedic and Trauma Unit, Department of Basic Medical Sciences, Neuroscience and Sense Organs, Spine Unit, University of Bari Aldo Moro, AOU Consorziale “Policlinico”, Bari, Italy; 3 Spine Surgery III, Scoliosis Department, IRCCS Orthopaedic Institute Galeazzi, Milan, Italy; 4 Tombolini Officine Ortopediche, San Giorgio Jonico, TA Italy; 5 Officine Ortopediche Maria Adelaide, Torino, Italy

**Keywords:** Scheuermann’s kyphosis, Maria adelaide brace, Anti-gravity brace, Developmental kyphosis, Bracing

## Abstract

**Purpose:**

This prospective observational study aims to assess the MA brace effectiveness in hyperkyphosis correction, focusing also on patients’ compliance of bracing and its psychological impact.

**Methods:**

Patients referring to our spine outpatient department with Scheuermann’s kyphosis (SK) from January 2011 to January 2017 were prospectively recruited. Patients were divided into two groups, according to their global thoracic kyphosis (TK): Group-A TK_T0_ < 60°, Group-B TK_T0_ ≥ 60°.

The MA brace was prescribed according to SRS criteria. Full spine X-rays were analyzed at conventional times: at the beginning of treatment (T0), at 6-months follow-up (T1, in-brace X-rays), at the end of treatment (T2) and at 2-year minimum follow-up from bracing removal (T3). At *T*_0_, *T*_2_ and *T*_3_ all the patients were assessed using the Italian Version of the SRS-22 Patient Questionnaire (I-SRS22). Variability between and within-groups was assessed; a *p* value < 0.05 was considered significant.

**Results:**

192 adolescents (87 girls and 105 boys, mean age 13.1) were recruited. The mean global TK at recruitment was 61.9° ± 11.3°, the mean follow-up time was 57.4 months. A good patients’ reported compliance was observed: 84.9% of patients used the brace as scheduled. A mean in-brace correction (in-brace TK_%_) of 37.4% was observed and a mean final correction (TK_%T3_) of 31.6%. At final follow-up (T3), curve reduction (ΔTK ≤  − 5°) was observed in 60.4% of patients and curve stabilization (− 5° < ΔTK < 5) in 29.7% of patients. At baseline, worse SRS22-mental health (*p* = 0.023) and self-image mean scores (*p* = 0.001) were observed in Group-B, compared with Group-A. At the end of treatment (T2), an improvement of all items was observed, wit significantly better improvement of self-image domain in Group-B.

**Conclusion:**

The MA brace has shown to be effective in the management of SK; good patients’ reported compliance and a positive effect on the patients’ mental status were recorded.

## Introduction

Scheuermann’s Kyphosis (SK) is a juvenile osteochondrosis of the spine characterized by defective growth of the vertebral cartilage endplate [1, 2]. It mainly involves thoracic (traditional pattern) or thoracolumbar spine. The onset of SK usually appears just before puberty, after ossification of the ring apophysis [3]. Adolescents affected by SK generally complain an excessive rigid thoracic or thoracolumbar hyperkyphosis, associated with concomitant vertebral structural changes and, sometimes, subacute thoracic pain [1, 2].

With a reported prevalence of 1–8% in the USA, SK currently represents the leading cause of sagittal spinal deformity in developmental age [2]. Although in the past SK was thought to be more frequent in male than female, recent reports have shown a comparable prevalence in both genders [3, 4].

SK is classically defined as a thoracic hyperkyphosis, greater than 45°, with three or more adjacent vertebrae wedged by at least 5° [5]; additional findings include wavy vertebral endplates, narrowing of the intervertebral disc space and presence of Schmorl’s nodes [6].

SK is considered an idiopathic disease, although mechanical factors together with a genetic predisposition have been implicated in its pathogenesis [7]. SK, indeed, may result from excessive mechanical stress on a weakened vertebral endplate during spine growth. The weakness of the vertebral endplate, on its turn, probably depends on an impaired, genetically determined quality of matrix components (collagen types II and IX) and chondrocytes [2, 7].

The management of SK includes both conservative treatment, most commonly with antigravity plaster casts or the Milwaukee brace (MB), and surgical options [4, 6, 8–15]; the patient’s age and the severity of the deformity are the main factors to be taken into account when choosing the management strategy [2, 16]. Bracing is currently the mainstay of treatment for SK, particularly before skeletal maturity and for less severe curves, since it has revealed effective in slowing or halting curve progression [2, 16–19].

Nonetheless, Huq et al. [16], in a recent systematic review have suggested that bracing provides less correction and might be less durable than surgery. This finding could be explained by assuming that social pressures might negatively impact on this kind of orthotic treatment, thus dissuading adolescents from using MB brace and creating compliance problems [2]: these could be the main reasons for insufficient control of adolescents’ hyperkyphosis by orthotic treatment [16, 20].

Therefore, the necessity of a brace that provides a similar curve correction, like that of MB, but allowing a higher patient’s compliance, thus reducing the psychological impact of bracing in adolescents, was clear and of increasing importance in the last decades.

In the current study, we describe an antigravity brace design, the Maria Adelaide (MA) brace, which is currently used at our institution as a gold standard for the orthotic treatment of SK. This study aims to assess the MA brace effectiveness in hyperkyphosis correction, focusing also on patients’ reported compliance of bracing and its psychological impact.

## Materials and methods

### Study subjects and clinical evaluation

Patients referring to our spine outpatient department with Scheuermann’s kyphosis (SK) from January 2011 to January 2017 were prospectively recruited. Ethical clearance was obtained from our centre’s clinical research ethics, as per the 1964 Declaration of Helsinki, and all patients and their parents gave informed consent before enrolment in the study.

192 adolescents (87 girls and 105 boys, mean age 13.1, range 9–14) were included in the present study.

Inclusion criteria: thoracic kyphosis (TK) greater than 45°; Risser score between 0 and 2 at baseline; apex T7 or lower; no contraindication to brace treatment.

Exclusion criteria: prior orthotic treatment with a different brace; TK greater than 75°; BMI > 25; concomitant scoliosis; concomitant musculoskeletal diseases; neurological diseases; metabolic diseases; the presence of congenital spine deformities; previous spinal surgery; concomitant disease able to alter the patient’s compliance.

Patients were divided into two groups, according to their kyphotic angle at recruitment (TK_T0_): Group-A with 45° ≤ TK_T0_ < 60° and Group-B with 60° ≤ TK_T0_ ≤ 75°.

Patients with TK > 50° were braced immediately and curves between 45° and 50° were braced if the progression of > 5° was found at 6-months follow-up.

The MA brace was prescribed according to SRS criteria; full-time use of the brace (i.e., 22 h per day) and daily physiotherapy practice was required. In patients with severe hyperkyphosis (i.e., TK > 70°), to obtain a greater correction of the deformity, an antigravity plaster cast was used for 40 days, before starting the treatment with MA brace.

### The Maria Adelaide brace

**Fig. 1:**
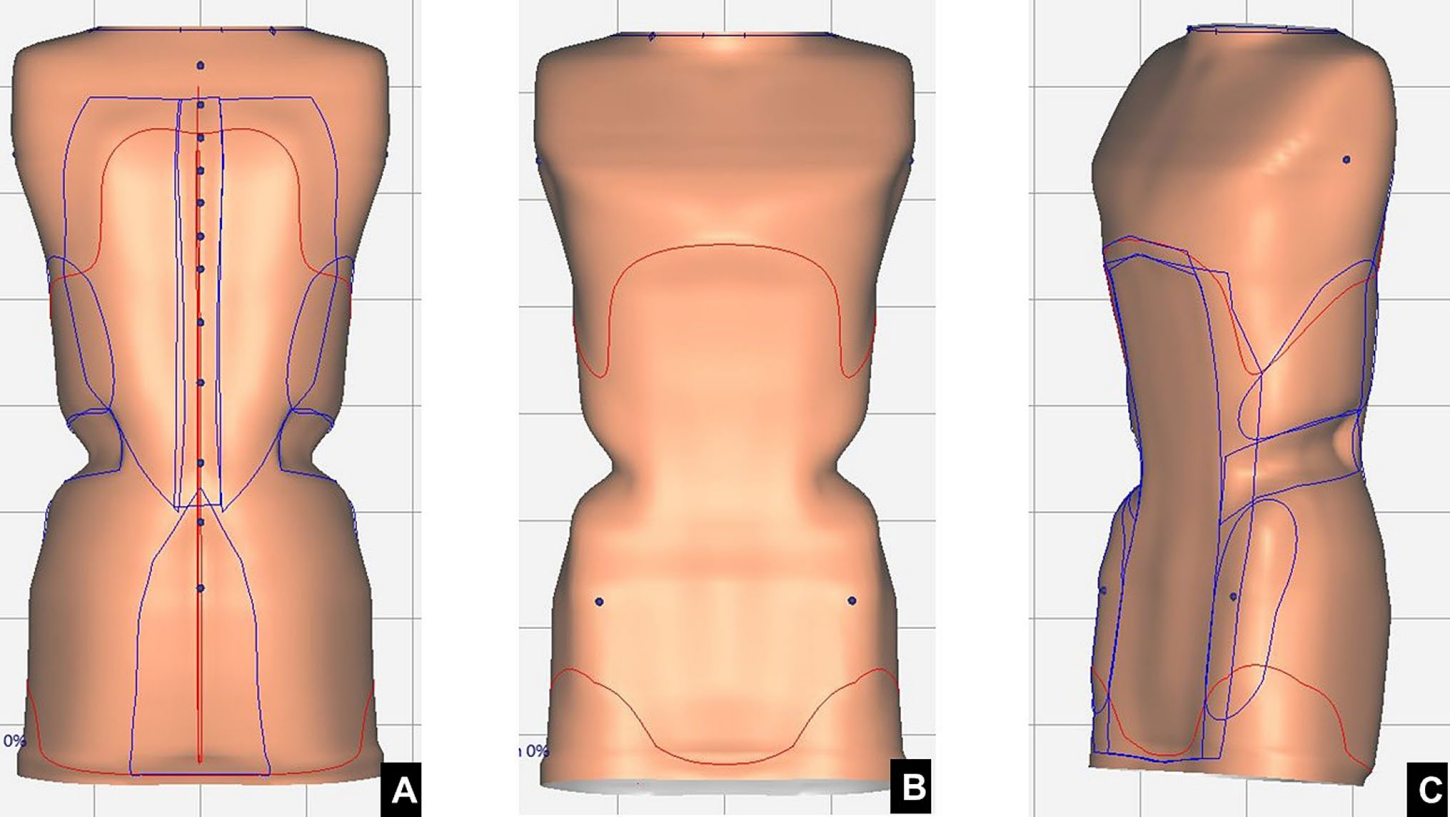
CAD/CAM MA brace model. **a** Anterior view. **b** Posterior view. **c** Lateral view

**Fig. 2:**
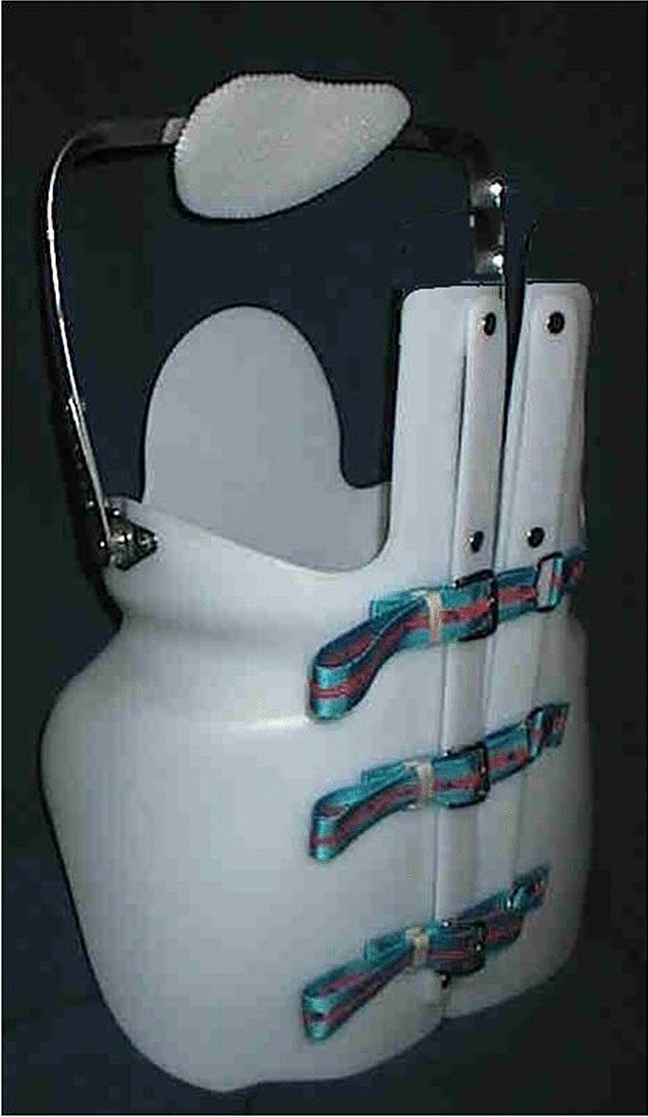
MA brace

**Fig. 3:**
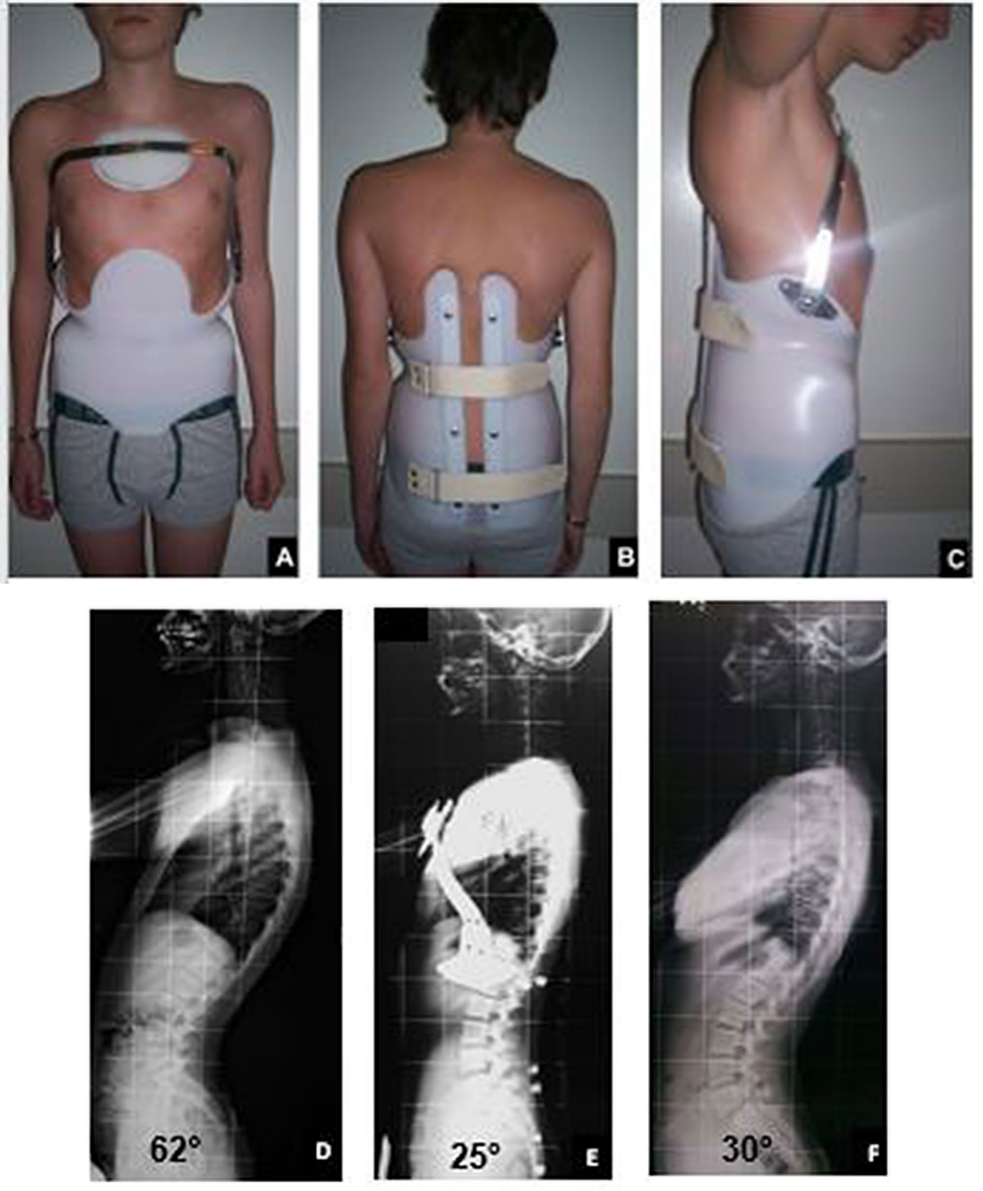
A 14-years old patient managed with MA brace. **a** Anterior view. **b** Posterior view. **c** Lateral view. **d** Lateral full spine X-rays at recruitment (*T*_0_). **e** Lateral full spine in-brace X-rays (*T*_1_). **f** Lateral full spine X-rays at the end of treatment (*T*_3_)

The MA brace was conceived in the early ‘90 s at “Maria Adelaide” Orthopaedic Institute, in Turin, by one of the senior Authors (MBB) and realized by his orthotic technician (AA), by modifying a preexisting brace. It is a univalve rigid brace, endowed with a sternal pad (Figs. 1, 2 and 3).

The biomechanical principles of this brace derived either from those applied for the construction of antigravity plaster cast on Risser frame, often used for the most severe cases and from those applied for the MB.

The thoracic kyphosis is mainly corrected by a forced reduction of lumbar lordosis and pelvic anteversion, induced by the pelvic girdle: its technical construction is the same of the MB, flattening the abdomen from the xiphoid process to pubis. Long posterior pads from buttocks to thoracic/thoracolumbar kyphosis are acting on its apex, and finally, the correction is stimulated by the anterior sternal pad, articulated on the pelvic girdle, and by postural self-corrective exercises.

The girdle is made of a 4–6 mm thick polyethylene, depending on the anthropometric characteristics of the patient. The girdle opening is placed dorsally, to flatten the lumbar lordosis. Otherwise, in less severe cases, a more comfortable ventral opening is possible, although less effective.

The sternal pad is connected to the girdle with two bilateral metal rods, thus allowing the correct adjustment of the sternal pad during the orthotic treatment.

Therefore, MA brace is based on the three-points biomechanical principle: the posterior force is applied at a level below the kyphotic curve apex, while the other two ventral forces are applied proximally below the suprasternal notch by the articulated pad and distally by flattening the compensatory lumbar lordosis and reducing pelvic anteversion.

### Clinical and radiological evaluation

At recruitment (*T*_0_), history and anthropometric data were recorded; upstanding full-spine anteroposterior and lateral X-rays were also performed.

All the patients underwent a 6-month clinical-radiographic evaluation, while the brace was checked at three months intervals. According to the study protocol, however, only the full spine X-rays performed at the following conventional times were analyzed: at the beginning of treatment (*T*_0_), at 6-months follow-up (*T*_1_, in-brace X-rays), at the end of treatment (*T*_2_) and at 2-year minimum follow-up from bracing removal (*T*_3_). In-brace X-rays were taken at *T*_1_, to check the corrective action of the MA brace. For other controls, all patients were asked to remove the brace 24 h before performing full-spine X-rays.

At each visit, moreover, patients and parents were asked about success or lack in scheduled wear of the brace. To best calculate the patients’ compliance, they were asked to keep diaries. At *T*_0_, *T*_2_ and *T*_3_ all the patients were assessed using the Italian Version of the SRS-22 Patient Questionnaire (I-SRS22).

The patients were treated until the end of bone growth (*T*_2_, average: 32.3 months), assessed on non-dominant wrist radiograph. If the deformity correction was obtained before reaching full skeletal maturity, maintenance treatment of 12–14 h per day was prescribed.

All radiographic measurements were obtained by two independent observers, using Surgimap (Nemaris Inc., Ver. 2.3.2, NY, USA), a validated software [21]; the interobserver concordance as assessed by the Cohen *K* statistic was high (0.9). Global TK (defined as T1–T12 angle; instead of T12, the last kyphotic vertebra was considered as lower end-vertebra in patients with thoracolumbar kyphosis), ΔTK (defined as TK_T0_–TK_T3_), in-brace correction (in-brace TK_%_ = TK_T0_—TK_T1_/TK_T0_ × 100) and final correction (TK_%_ = TK_T0_—TK_T3_/TK_T0_ × 100) were assessed.

### Statistical analysis

Statistical analysis was carried out using SPSS^®^ (version 23; IBM Corp, Armonk, NY). The Shapiro–Wilk test was conducted to verify the normal distribution of the data. Unpaired *t* test and Chi-square test were performed to assess intergroup variability; paired *t* test was performed to assess within-group variability.

Finally, results were analyzed according to ΔTK; three possible outcomes were assessed: curve correction (ΔTK ≤  − 5°), curve stabilization (− 5° < ΔTK < 5°) and curve progression (ΔTK ≥ 5°). All tests were two-sided, with significance; a *p* value of less than 0.05 was considered significant.

## Results

The main data of the study are summarized in Table 1. The mean global TK_T0_ at recruitment was 61.9° ± 11.3°, the mean follow-up time was 57.4 months (Table 1). In five patients out of 192 (2.6%), an antigravity plaster cast was used for forty days, before starting the treatment with MA brace. Good compliance was observed: 163 patients out of 192 (84.9%) used the brace as scheduled. Moreover, significant higher compliance was observed in Group-B patients (*p* = 0.022; Table 2).

**Table 1:** Main data of the study

	All (*n* = 192)	Group-A 45° ≤ TK_T0_ < 60° (*n* = 76)	Group-B 60 ≤ TK _T0_ ≤ 75° (*n* = 116)
Gender			
Female (%)	105 (54.7%)	41 (53.9%)	64 (55.2%)
Age			
Mean ± SD	13.1 ± 2.8	12 ± 2.1	14 ± 2.9
Range	9—15	10–13	9–15
Skeletal age (at recruitment)			
Risser 0, *n* (%)	86 (44.8%)	35 (46.1%)	51 (44%)
Risser 1, *n* (%)	65 (33.8%)	31 (40.8%)	34 (29.3%)
Risser 2, *n* (%)	41 (21.3%)	10 (13.2%)	31 (26.7%)
Global TK_T0_			
Mean ± SD	61.9 ± 11.2	55.7 ± 4.5	66.3 ± 7.9
Range	49°–73°	49°–59°	60°–73°
T vertebrae, *n* (mean ± SD)	12.1 ± 1.5	12.1 ± 0.9	12.2 ± 1.9
Compliance data			
Compliant patients, *n* (%)	163 (84.9%)	58 (76.3%)	105 (90.5%)
Non-compliant patients, *n* (%)	29 (15.1%)	18 (23.7%)	11 (9.5%)
Brace refusal, *n* (%)	0	0	0
Brace with posterior opening, *n* (%)	160 (83.3%)	46 (60.5%)	114 (98.3%)
Mean follow-up time (months, mean ± SD)	57.3 ± 5.6	59.6 ± 6.3	55.2 ± 4.4

**Table 2:** Radiographic evaluation and compliance data: differences between groups (Group-A vs Group-B)

	All (Mean ± SD)	Group-A 45° ≤ TK_T0_ < 60° (Mean ± SD)	Group-B 60° ≤ TK_T0_ ≤ 75° (Mean ± SD)	*p*
TK_T0_	61.9 ± 11.2	55.7 ± 4.5	66.3 ± 7.9	0.003^*^
TK_T1_ (in-brace)	37.5 ± 9.1	35.8 ± 5.6	39.3 ± 8.3	0.087
TK_T2_	38.9 ± 7.3	37.8 ± 6.2	41.5 ± 6.8	0.0625
TK_T3_	41.7 ± 4.4	40.3 ± 7.6	43.3 ± 5.3	0.0923
ΔTK	19.5 ± 5.8	15.5 ± 4.8	23.1 ± 3.6	0.021*
In-brace TK_%_	37.4%	35.1%	40.7%	**0.01**^**§**^
TK_%T3_	31.6%	27.7%	34.7%	**0.005**^**§**^
Compliant patients (%)	84.9%	76.3%	90.5%	**0.022**^**§**^
Curve reduction: ΔTK ≤ − 5° (%)	116 (60.4%)	45 (59.2%)	71(61.2%)	0.526
Curve stabilization: − 5° < ΔTK < 5 (%)	57 (29.7%)	24 (31.6%)	33 (28.5%)	0.065
Curve progression: ΔTK ≥ 5° (%)	19 (9.9%)	7 (9.2%)	12 (10.4%)	0.773

*Significant *p* value (unpaired *t* test)

^§^Significant *p* value (Chi-square test)

Table 2 summarizes radiographic and compliance differences between groups. A mean in-brace correction (in-brace TK_%_) of 37.4% was observed and a mean final correction (TK_%T3_) of 31.6%; in Group-B a significant higher in-brace TK_%_ (*p* = 0.005) and mean TK_%T3_ (*p* = 0.022). At final follow-up (*T*_3_), curve reduction (ΔTK ≤  − 5°) was observed in 60.4% of patients and curve stabilization (− 5° < ΔTK < 5) in 29.7% of patients.

Table 3 shows the paired *t* test *p* values for global TK differences within groups at each follow-up (TK_T1_; TK_T2_; TK_T3_) versus baseline (TK_T0_).

**Table 3:** Paired *t* test *p* values for Global TK differences within groups at each follow-up (TK_T1_; TK_T2_; TK_T3_) versus baseline (TK_T0_)

	All	Group-A 45° ≤ TK_T0_ < 60°	Group-B 60 ≤ TK _T0_ ≤ 75°
TK_T1_ (in-brace)	< 0.001*	< 0.001*	< 0.001*
TK_T2_	< 0.001*	< 0.001*	0.001*
TK_T3_	< 0.001*	< 0.001*	0.003*

*Significant *p* value (paired *t* test)

**Fig. 4:**
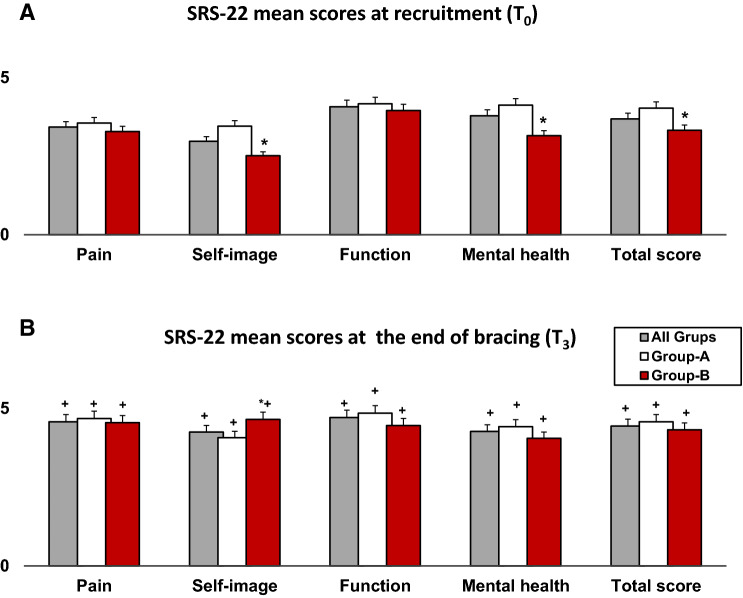
SRS-22 mean scores at recruitment (Fig. 1a) and at the end of bracing (Fig. 1b). *p < 0.05, *p* values indicate differences between groups (unpaired *t* test); ^+^*p* < 0.05, *p* values indicate differences within groups (baseline vs end of bracing; paired *t* test)

Figure 3 shows a 14 years old patient managed with MA brace. Figure 4 shows SRS-22 mean scores at recruitment (*T*_0_; Fig. 4a) and at the end of bracing (*T*_2_; Fig. 4b); at baseline, worse SRS22-mental health (*p* = 0.023) and self-image mean scores (*p* = 0.001) were observed in Group-B, compared with Group-A. At the end of treatment (*T*_2_), an improvement of all items was depicted; however, a significantly better improvement of self-image domain was observed in Group-B.

**Fig. 5:**
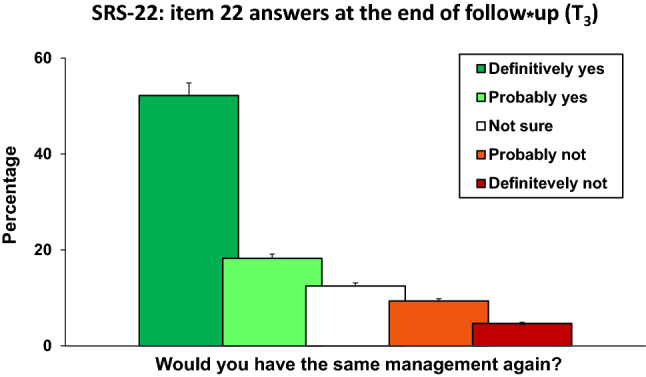
Answers to question 22 from the SRS-22 questionnaire recorded at the end of follow-up (*T*_3_): “Would you have the same management again if you had the same condition?”

Figure 5 shows the answers to the question “Would you have the same management again if you had the same condition?” (item 22 from SRS-22 questionnaire) recorded at the end of follow-up (*T*_3_): 52.2% of patients answered, “definitively yes” and 18.2% “probably yes”.

## Discussion

SK is the most prevalent cause of structural hyperkyphosis in adolescents, thus causing back pain and body image dissatisfaction during developmental age [2, 10, 16]. It is also reported that curves greater than 65° may continue to progress even after skeletal maturity, thus leading in adulthood to very severe deformity, chronic back pain, lower quality of the life and poorer general health, compared with the general population [2, 10, 22–24].

Orthotic treatment of Scheuermann’s Kyphosis (SK) is indicated for painful and/or mild kyphosis (45° < TK° < 65°), while its efficacy in the management of severe kyphosis, greater than 70°, still matters of debate [2, 19, 25]. In European spinal deformity centres, therefore, patients referring with a hyperkyphosis ≥ 70° are generally treated with an antigravity plaster cast for forty days, before starting using a polyethylene-made brace.

Different types of brace have been proposed in the management of SK, including Milwaukee brace, plaster brace and antigravity brace. The efficacy of Milwaukee brace in the management of SK relies on very robust data. Originally used by Bradford et al. [19] and further investigated in several studies [10, 22, 23, 26], the Milwaukee brace resulted effective in the management of SK, showing a curvature reduction by 35–50%, at 32 months follow-up [2, 19, 23, 26]. Moreover, at 5 years follow-up 66% of patients showed an improvement of the initial curvature and 10% of patients presented a stable curve [2, 19, 23, 26].

Nonetheless, patients’ compliance is the main concern of Milwaukee brace treatment, mainly because of the aesthetic and psychological impact of the neck ring [2, 26, 27].

To improve the patients’ compliance and reduce the psychological impact of bracing, antigravity braces, based on the biomechanical action of the three points principle, have been developed [25, 28, 29]. This prospective observational study aims to assess the effectiveness and the psychological impact of the MA brace in a large cohort.

In the present study, we recruited 192 adolescents (87 girls and 105 boys, mean age 13.1); according to their global kyphosis at recruitment (TK_0_), patients were divided into two groups: 76 patients out of 192 (39.6%) had 45° ≤ TK_T0_ < 60° (Group-A), while the remaining 116 patients (60.4%) showed 60 ≤ TK_T0_ ≤ 75° (Group-B). All the patients performed the scheduled follow-up, including non-compliant patients, and no patients needed surgery at the end of follow-up.

Good compliance was observed in the use of the MA brace: the overall compliance rate was 84.9% and the compliance rate recorded in Group-B patients was higher than in Group-A (*p* = 0.022). As shown in Fig. 5, at the question “Would you have the same management again if you had the same condition?” (item 22 from SRS-22 questionnaire), more than half of patients (52.21%) answered “definitively yes” and 18.2% answered, “probably yes”.

It is important to remark Group-B patients showed worse SRS22-mental health (*p* = 0.023) and self-image mean scores at recruitment (*p* = 0.001), so these data might also explain the higher compliance rate recorded in patients with more severe deformity (Fig. 4).

A higher patients’ reported compliance was recorded in the use of MA brace when compared with Milwaukee brace [19, 22, 23, 26, 27]. The higher reported compliance observed in the use of antigravity braces, confirmed in previous studies [17, 28, 29], could depend on the short brace design, that could be easily hidden under a large t-shirt or sweater.

At the end of follow-up, curve reduction was observed in 60.4% of patients and curve stabilization in 29.7% of adolescents (Table 1). In Group-B patients, a significantly higher final correction (TK%_T3_) was depicted (*p* = 0.005). This finding could depend both on the higher compliance observed in patients with more severe deformity, and on the more effective anteroinferior pressure area in girdles with a posterior opening. Therefore, skeletally immature patients with a more severe TK and a marked compensative hyperlordosis, treated with an MA brace with posterior opening, could benefit from a greater curve improvement. Moreover, it is also reported the pelvic girdle plays a key-role in hyperkyphosis correction, by reducing pelvic anteversion and flattening lumbar lordosis, thus providing a more effective correction of the deformity [27].

The effectiveness of antigravity braces in the management of SK was also shown in previous studies conducted using braces with a similar design [28, 29]. However, in the current study, we describe a different type of antigravity brace, more similar to the effect of MB pelvic girdle and the Risser antigravity plaster casts, and we assess its efficacy in a larger patients’ cohort, also focusing on their mental health and their acceptance of this brace.

The main limitation of this study is the lack of a control group. However, in patients meeting the inclusion and exclusion criteria described in the present study we do not prescribe anymore MB, because of the very low patients’ compliance and parents’ acceptance. Therefore, we are not able to perform a randomized controlled trial comparing MA brace to Milwaukee brace. Although we recruited a quite big number of patients, the lack of a power analysis is another limitation of the present study. Finally, we were not able to exactly record the patients’ compliance in the use of the MA brace—for instance with a pressure or a temperature monitor—hence we only recorded the patients’ reported compliance.

We hypothesize that, if the present data will be confirmed in different countries and different social contexts, in a future multicenter study, for instance, the MA brace could definitively become the more valid alternative to MB in the management of SK, as it happened in our Centers, and it could be successfully applied also in postural round back deformities on a part-time basis.

Furthermore, although going beyond the aims of the present study, we think it is useful to remark MA antigravity brace could be also used part-time, together with rehabilitation, in the management of postural hyperkyphosis.

## Conclusion

The MA brace has revealed clinically and radiographically effective in the conservative management of SK, thus avoiding surgical treatment in patients with global TK < 75°.

Moreover, good patients’ compliance and a positive effect on the SRS22-mental health score and SRS22-self-image score was observed at the final follow-up (*T*_2_).

Based on these findings, we suggest the use of this type of antigravity brace in the management of SK, as well as for non-structural adolescent postural round back, with still better outcomes.

## References

[1] Lowe TG (1990). Scheuermann disease. J Bone Jt Surg.

[2] Palazzo C, Sailhan F, Revel M (2014). Scheuermann’s disease: an update. Jt Bone Spine.

[3] Lowe TG, Line BG (2007). Evidence based medicine: analysis of Scheuermann kyphosis. Spine.

[4] Faldini C, Traina F, Perna F (2015). Does surgery for Scheuermann kyphosis influence sagittal spinopelvic parameters?. Eur Spine J.

[5] Sørensen KH (1964). Scheuermann’s Juvenile Kyphosis.

[6] Lamartina C (2010). Posterior surgery in Scheuermann’s kyphosis. Eur Spine J.

[7] Zaidman AM, Zaidman MN, Strokova EL (2013). The mode of inheritance of scheuermann’s disease. Biomed Res Int.

[8] Ghasemi A, Stubig T, Nasto LA (2017). Distal junctional kyphosis in patients with Scheuermann’s disease: a retrospective radiographic analysis. Eur Spine J.

[9] Gennari JM, Aswad R, Ripoll B, Bergoin M (1997). Indications for surgery in so-called “regular” thoracic and thoracolumbar kyphosis. Eur Spine J.

[10] Etemadifar M, Ebrahimzadeh A, Hadi A, Feizi M (2016). Comparison of Scheuermann’s kyphosis correction by combined anterior–posterior fusion versus posterior-only procedure. Eur Spine J.

[11] Behrbalk E, Uri O, Parks RM (2014). Posterior-only correction of Scheuermann kyphosis using pedicle screws: economical optimization through screw density reduction. Eur Spine J.

[12] Guler O, Akgul T, Korkmaz M (2017). Postoperative changes in sacropelvic junction in short-segment angular kyphosis versus Scheuermann kyphosis. Eur Spine J.

[13] Koller H, Juliane Z, Umstaetter M (2014). Surgical treatment of Scheuermann’s kyphosis using a combined antero-posterior strategy and pedicle screw constructs: efficacy, radiographic and clinical outcomes in 111 cases. Eur Spine J.

[14] Yun C, Shen CL (2017). Anterior release for Scheuermann’s disease: a systematic literature review and meta-analysis. Eur Spine J.

[15] Piazzolla A, Montemurro V, Bizzoca D, Parato C, Carlucci S (2019). Accuracy of plain radiographs to identify malpositioned free hand pedicle screw in the deformed spine. J Neurosurg Sci.

[16] Huq S, Ehresman J, Cottrill E (2020). Treatment approaches for Scheuermann kyphosis: a systematic review of historic and current management. J Neurosurg Spine.

[17] de Mauroy JC, Weiss HR, Aulisa AG (2010). 7thSOSORT consensus paper: conservative treatment of idiopathic and Scheuermann’s kyphosis. Scoliosis.

[18] Zaina F, Atanasio S, Ferraro C (2009). Review of rehabilitation and orthopedic conservative approach to sagittal plane diseases during growth: hyperkyphosis, junctional kyphosis, and Scheuermann disease. Eur J Phys Rehabil Med.

[19] Bradford DS, Moe JH, Montalvo FJ, Winter RB (1974). Scheuermann’s kyphosis and roundback deformity. Results of Milwaukee brace treatment. J Bone Jt Surg Ser A.

[20] Rahman T, Bowen JR, Takemitsu M, Scott C (2005). The association between brace compliance and outcome for patients with idiopathic scoliosis. J Pediatr Orthop.

[21] Piazzolla A, Solarino G, Bizzoca D (2018). Spinopelvic parameter changes and low back pain improvement due to femoral neck anteversion in patients with severe unilateral primary hip osteoarthritis undergoing total hip replacement. Eur Spine J.

[22] Farsetti P, Tudisco C, Caterini R, Ippolito E (1991). Juvenile and idiopathic kyphosis—long-term follow-up of 20 cases. Arch Orthop Trauma Surg.

[23] Montgomery SP, Erwin WE (1981). Scheuermann’s kyphosi—long-term results of Milwaukee brace treatment. Spine (Phila Pa 1976).

[24] Ristolainen L, Kettunen JA, Heliövaara M (2012). Untreated Scheuermann’s disease: a 37-year follow-up study. Eur Spine J.

[25] Gutowski WT, Renshaw TS (1988). Orthotic results in adolescent kyphosis. Spine (Phila Pa 1976).

[26] Sachs B, Bradford D, Winter R (1987). Scheuermann kyphosis. Follow-up of Milwaukee-brace treatment. J Bone Jt Surg Ser A.

[27] Etemadifar MR, Jamalaldini MH, Layeghi R (2017). Successful brace treatment of Scheuermann’s kyphosis with different angles. J Craniovertebr Junction Spine.

[28] Aulisa AG, Falciglia F, Giordano M (2016). Conservative treatment in Scheuermann’s kyphosis: comparison between lateral curve and variation of the vertebral geometry. Scoliosis Spinal Disord.

[29] Weiss HR, Turnbull D, Bohr S (2009). Brace treatment for patients with Scheuermann’s disease—a review of the literature and first experiences with a new brace design. Scoliosis.

